# Antifungal Activity of *Bacillus* Species Against *Fusarium* and Analysis of the Potential Mechanisms Used in Biocontrol

**DOI:** 10.3389/fmicb.2018.02363

**Published:** 2018-10-02

**Authors:** Noor Khan, Pilar Martínez-Hidalgo, Tyler A. Ice, Maskit Maymon, Ethan A. Humm, Najmeh Nejat, Erin R. Sanders, Drora Kaplan, Ann M. Hirsch

**Affiliations:** ^1^Departments of Molecular, Cell, and Developmental Biology, University of California, Los Angeles, Los Angeles, CA, United States; ^2^Center for Education Innovation and Learning in the Sciences, University of California, Los Angeles, Los Angeles, CA, United States; ^3^Department of Environmental Hydrology and Microbiology, Zuckerberg Institute for Water Research, Jacob Blaustein Institutes for Desert Research, Ben-Gurion University of the Negev, Beersheba, Israel; ^4^Molecular Biology Institute, University of California, Los Angeles, Los Angeles, CA, United States

**Keywords:** plant growth promoting bacteria, biocontrol bacteria, *Bacillus*, *Fusarium*, hydrolytic enzymes

## Abstract

*Fusarium* is a complex genus of ascomycete fungi that consists of plant pathogens of agricultural relevance. Controlling *Fusarium* infection in crops that leads to substantial yield losses is challenging. These economic losses along with environmental and human health concerns over the usage of chemicals in attaining disease control are shifting focus toward the use of biocontrol agents for effective control of phytopathogenic *Fusarium* spp. In the present study, an analysis of the plant-growth promoting (PGP) and biocontrol attributes of four bacilli (*Bacillus simplex* 30N-5, *B. simplex* 11, *B. simplex* 237, and *B. subtilis* 30VD-1) has been conducted. The production of cellulase, xylanase, pectinase, and chitinase in functional assays was studied, followed by *in silico* gene analysis of the PGP-related and biocontrol-associated genes. Of all the bacilli included in this study, *B. subtilis* 30VD-1 (30VD-1) demonstrated the most effective antagonism against *Fusarium* spp. under *in vitro* conditions. Additionally, 100 μg/ml of the crude 1-butanol extract of 30VD-1’s cell-free culture filtrate caused about 40% inhibition in radial growth of *Fusarium* spp. Pea seed bacterization with 30VD-1 led to considerable reduction in wilt severity in plants with about 35% increase in dry plant biomass over uninoculated plants growing in *Fusarium*-infested soil. Phase contrast microscopy demonstrated distortions and abnormal swellings in *F. oxysporum* hyphae on co-culturing with 30VD-1. The results suggest a multivariate mode of antagonism of 30VD-1 against phytopathogenic *Fusarium* spp., by producing chitinase, volatiles, and other antifungal molecules, the characterization of which is underway.

## Introduction

Numerous species of *Bacillus* have been identified as plant-growth promoting bacteria (PGPB; Bashan and Holguin, 1998) and/or biocontrol agents (BCA) (Compant et al., 2005; Khan et al., 2017). The most commonly studied PGPB/BCA are *B. amyloliquefaciens, B. licheniformis*, and *B. subtilis.* PGPB employ a variety of strategies to enhance plant growth and survival by both direct and indirect mechanisms. The most common direct mechanisms are phytohormone production, the acquisition of nutrients such as phosphorous and nitrogen, and the control of pathogens through various means, for example, through the synthesis of hydrolytic enzymes, antifungal compounds, lipopeptides, or antibiotics. The indirect mechanisms include protection from abiotic stress brought about by drought, salinity, etc., the triggering of specific defense-related pathways, particularly the induction of systemic resistance (ISR) against pathogens and pests (Khan et al., 2012; Martínez-Hidalgo et al., 2015); and the release of volatile organic compounds [VOCs; also called bacterial volatile compounds (BVCs; Bernier et al., 2011) as well as the suppression of reactive oxygen species (ROS)]. Numerous articles and reviews about the variety of mechanisms PGPB use to promote plant growth have been published (de Boer et al., 2003; Vessey, 2003; Lugtenberg and Kamilova, 2009; Glick, 2012; Yuan et al., 2012; Ramyabharathi and Raguchander, 2014; Goswami et al., 2016).

Recently, *B. simplex* has been added to the growing list of PGPB (Ertruk et al., 2010; Hassen and Labuschagne, 2010; Schwartz et al., 2013; Velivelli et al., 2015). Prior to these studies, *B. simplex* was known mostly for its slope-specific adaptation and incipient sympatric speciation in “Evolution Canyons I and II” in Israel (Sikorski and Nevo, 2005). Earlier, we described several of the traits required for a number of the mechanisms for both plant-growth promotion and biocontrol in *B. simplex* 30N-5, including evidence for the synthesis of polyamines, the production of siderophores, and the synthesis of antimicrobial peptides and antibiotics (Maymon et al., 2015). *B. simplex* 30N-5 also has ACC (1-aminocyclopropane-1-carboxylic acid) deaminase activity, based on the ability of *B. simplex* 30N-5 to grow on ACC (Schwartz et al., 2013). In this report, we compared *B. simplex* 30N-5 and *B. subtilis* 30VD-1, both isolated from the UCLA Mildred E. Mathias Botanical Garden (Schwartz et al., 2013). The other two *B. simplex* strains, 237 and 11, were isolated from the Negev Desert in Israel (Kaplan et al., 2013). Our earlier study (Schwartz et al., 2013) showed that *B. simplex* 30N-5 inhibits the growth of two members of the *F. oxysporum* complex and *F. solani*, formerly *Nectria haematococca* (Geiser et al., 2013). However, still little is known about the effectiveness of *B. simplex* strains in terms of their usefulness as PGPB/BCA.

Mechanisms for both direct and indirect PGPB functions are described through the use of physiological and biochemical assays, and more recently by *in silico* analyses. Although not all of the genomes of *B. simplex* strains are sequenced, the genome sequences of eight are available including those analyzed here: *B. simplex* strain 30N-5 (Maymon et al., 2015) and also strains II3b11 (Sikorski and Nevo, 2005), P558 (Croce et al., 2014), and BA2H3 (Khayi et al., 2015). Having genome sequences from a diverse set of *B. simplex* strains from different habitats presents an excellent opportunity to learn more about the potential BCA of this species.

*Fusarium* is an ascomycete that causes plant infections known as head blights, vascular wilts, patch diseases, root rots, and yellowing disease. Although *Fusarium* fungi can invade plants through seeds and wounds, they also enter plants through their roots (Lagopodi et al., 2002; Aydi Ben Abdallah et al., 2016), especially if root cap border cells are damaged (Gunawardena and Hawes, 2002). The disease is managed by crop rotation, planting resistant crops, letting the land lie fallow, and/or applying chemical fungicides. Because fungicides have negative effects on both the environment and human health and, in addition, can promote the development of resistant strains of certain disease-causing fungi (Holmes and Eckert, 1999), efforts to utilize plant-associated bacteria to manage *Fusarium*-induced pathogenesis have been pursued. Most of the bacteria studied for biocontrol activity are pseudomonads and species of *Bacillus*, which use diverse mechanisms for the biocontrol of *Fusarium*, including siderophore-mediated competition for iron, antibiotic production, and induced systemic resistance (Raaijmakers et al., 1995).

In this report, we tested the potential PGP activity of an understudied *Bacillus* species and a *B. subtilis* strain for demonstrable functions that correlated with the *in silico* analysis of plant-growth promotion-related and biocontrol-associated genes. *B. simplex* 30N-5, 11, and 237, and also *B. subtilis* 30VD-1, were compared to the well-known PGP bacilli, such as *B. pumilus, B. amyloliquefaciens*, and *B. licheniformis*. Employing a comparative approach between *in silico* studies and a demonstrable chemical product or activity is likely to lead to a more rational choice of probiotic microbes for promoting plant growth and inhibiting disease.

## Results

### *Bacillus–Fusarium* Interaction

**FIGURE 1:**
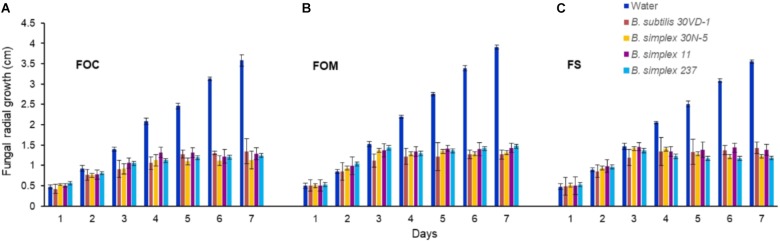
Average of the measurements of fungal radial growth toward the bacterial strain after 7 days of incubation compared to the control. All four *Bacillus* strains (*B. subtilis* 30VD-1, *B. simplex* 30N-5, *B. simplex* 11, and *B. simplex* 237) inhibited the growth of *Fusarium* spp. **(A)** *F. oxysporum* f. sp. *conglutinans* (FOC), **(B)** *F. oxysporum* f. sp. *matthioli* (FOM), **(C)** *F. solani* (FS). Values presented as mean ± standard error. The experiment was repeated 6 times with 10 replicates in each trial.

We compared *in vitro* antagonism of *B. simplex* 30N-5 and *B. subtilis* 30VD-1 on *Fusarium* spp. to that elicited by the Negev-desert isolated *B. simplex* strains, 11 and 237. All four bacterial strains prevented *F. oxysporum* f. sp. c*onglutinans* (FOC), *F. oxysporum* f. sp. *matthioli* (FOM), and *F. solani* (FS) from forming dense hyphal mats with similar efficiency (**Figure 1**). After 7 days of incubation, the four different bacteria had inhibited fungal growth by 60–70% in comparison to the controls (**Figure 1**). Although *B. simplex* 30N-5, 11, and 237 each caused extensive hyphal thinning and a comparatively less dense hyphal network than the fungus in the control set, they were unable to halt FOM, FOC, and FS growth as markedly as did *B. subtilis* 30VD-1. Phase contrast microscopy of the fungal mycelia in confrontation assays with *B. subtilis* 30VD-1 revealed distortions and abnormal swelling resulting in bulbous structures in the hyphal cell wall. No such changes were detected in control mycelia (**Supplementary Figure S1**).

### Genome Analysis and Functional Assays for Hydrolytic Enzymes

Because of the hyphal thinning phenotype, we hypothesized that growth inhibition might be brought about by the activity of hydrolytic enzymes. Although the genome of *B. simplex* 30N-5 is in permanent draft stage (IMG genome ID: 2516653086), it allowed us to detect genes, which when expressed, could be involved in inhibiting fungal growth. The fact that additional *B. simplex* genomes are available made them useful as scaffolds to find missing genes in one or more of the species.

### Chitin-Degrading Enzymes

*Fusarium* cell walls are composed of chitin, α-1,3-glucans, and β-1,3-glucans (Schoffelmeer et al., 1999), suggesting that chitin-degrading enzymes and/or glucanases are likely to be responsible for *B. subtilis* 30VD-1’s effects on hyphal thinning. Because we lack a complete genome sequence for *B. subtilis* 30VD-1, we used the *B. simplex* genomes to elucidate the identity of the genes responsible for cell wall degradation. The 30VD-1 genome sequence is in draft stage and undergoing analysis. However, preliminary studies strongly suggested that many of the genes detected in the *B. simplex* genomes are found in *B. subtilis* 30VD-1.

Two distinct mechanisms are known for degrading chitin, a linear polymer of β-1,4-linked N-acetylglucosamine (NAG) residues, which shares structural similarities with cellulose (Beier and Bertilsson, 2013; Chavan and Deshpande, 2013). One mechanism employs endochitinases, also known as 1,4-β-poly-N-acetylglucosaminidases (EC 3.2.1.14). Exochitinases, cellobiase/β-glucosidases (EC 3.2.1.21), or exoglucanases, now classified as β-N-acetylhexosaminidases (*nagZ*) (EC 3.2.1.52), release NAG dimers and monomers from chitin (Horsch et al., 1997). Although we found no evidence for genes encoding 1,4-β-poly-N-acetylglucosaminidase or any other chitinase (*chiC* or *chiA*) or similar genes in the sequenced *B. simplex* strains except for *B. simplex* VanAntwerpen02 (data not shown), the *nagZ* gene and two genes designated as 6-phospho-β-glucosidase (*celF*) were detected in the *B. simplex* genomes, with high identity among them (orange shading, **Supplementary Figure S2**). The *B. simplex nagZ* genes are only 62–69% identical to *nagZ* in the more typical PGP bacilli (blue shading, **Supplementary Figure S2**), and show 80–98% identity among the eight sequenced *B. simplex* strains. Furthermore, neither *B. simplex* 30N-5 nor *B. subtilis* 30VD-1 were positive in tests using the chitin azure assay, which detects chitinase activity, whereas *Paenibacillus tundrae* 47 was positive (Kaplan et al., 2013).

**FIGURE 2:**
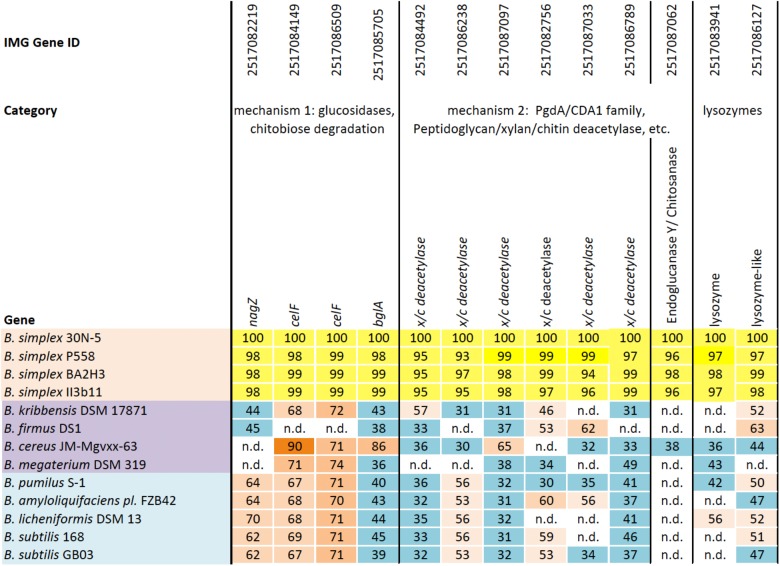
Homologs of a reference set of genes likely to be involved in inhibiting fungal growth through hydrolytic enzyme production. Top row, IMG gene ID number; second row, general categories of biocontrol; third row, genes identified in each *Bacillus* strain (left column); *B. simplex* strains (orange); *B. cereus/megaterium* group (purple); and typical PGP bacilli (blue). The percentages of gene identity are included within the individual cells. Sequence alignments were based on blastp searches (% values in cells). Varying shades of orange represent percent identities to *B. simplex* 30N-5 (set to 100%): ≥90%, dark orange; 71–89%, medium orange; 60–70%, light orange; 50–59%, pale orange. The turquoise-blue cells represent sequence identities of ≤49%. Genes not detected, n.d.

The identity of the two *B. simplex celF* genes compared to the *B. firmus/B. cereus/B. megaterium* group of *Bacillus* spp. in our survey (purple shading, **Supplementary Figure S2**), varied from 71 to 74% for *B. megaterium* and 90% to the *B. cereus* JM-mgvxx-63 gene. A *celF* ortholog in *B. kribbensis* DSM 17871 was 68–72% identical to the *B. simplex* genes, but no *celF* ortholog was detected in *B. firmus* DS1. A third 6-phospho-β-glucosidase, annotated in several *Bacillus* species as a broad-specificity cellobiase (*bglA*), was 86% identical to a gene in *B. cereus* JM-mgvxx-63 (**Figure 2**), but we saw no evidence in the *B. simplex* genomes for genes encoding cellobiase or cellulase except for IMG2517085743, which was annotated as Cellulase M (an endoglucanase; EC:3.2.1.4). Evidence that this gene could encode cellulase is suggested by the hydrolytic enzyme assay, in which the *B. simplex* and the *B. subtilis* strains formed halos on CMC plates (**Table 1** and **Supplementary Figure S2**). Of the four strains, however, *B. subtilis* 30VD-1 produced the largest halo on CMC plates compared to its colony size (**Supplementary Figure S2**). A cellulase (glycosyl hydrolase family 5) gene was detected in the incomplete 30VD-1 genome, but it exhibited very little sequence similarity with the *B. simplex* genes.

**Table 1:** Summary of relative hydrolytic enzyme activities exhibited by three different *Bacillus simplex* strains and *B. subtilis* 30VD-1.

Bacterial species	Chitinase activity	Cellulase activity	Pectinase activity	Xylanase activity	Protease activity
*B. simplex* 30N-5	+	+	+	+	+
*B. simplex* 237	+	+	+	+	+
*B. simplex* 11	+	+	+	+	+
*B. subtilis* 30VD-1	++	+++	++	+	+
*P. tundrae* 47	+++	n.t.	n.t.	n.t.	n.t.
*K. rosea* 245	−	n.t.	n.t.	n.t.	n.t.

*Paenibacillus tundrae 47 and Kocuria rosea 245 were the positive and negative controls, respectively, for the chitinase activity plates. n.t., not tested.*

The second mechanism of chitin breakdown requires deacetylation of chitin through the activity of chitin deacetylases (CDA; EC 3.5.1.41) followed by subsequent hydrolysis of the deacylated chitin by chitosanase (EC 3.2.1.132) (Beier and Bertilsson, 2013). Seven different sequences in the *B. simplex* 30N-5 genome were annotated as xylan/chitin- or peptidyl/chitin-deacetylases and six of them are shown in **Figure 2**. A gene sequence annotated as endoglucanase Y/chitosanase was detected in *B. simplex* 30N-5 and the other *B. simplex* genomes (**Figure 2**). This same gene, called endoglucanase Y in *B. cereus* JM-Mgvxx-63, is only 38% identical to the *B. simplex* 30N-5 gene. Similarly, a chitosanase gene was detected in *B. subtilis* 30VD-1, albeit at a very low identity to the *B. simplex* genes.

**FIGURE 3:**
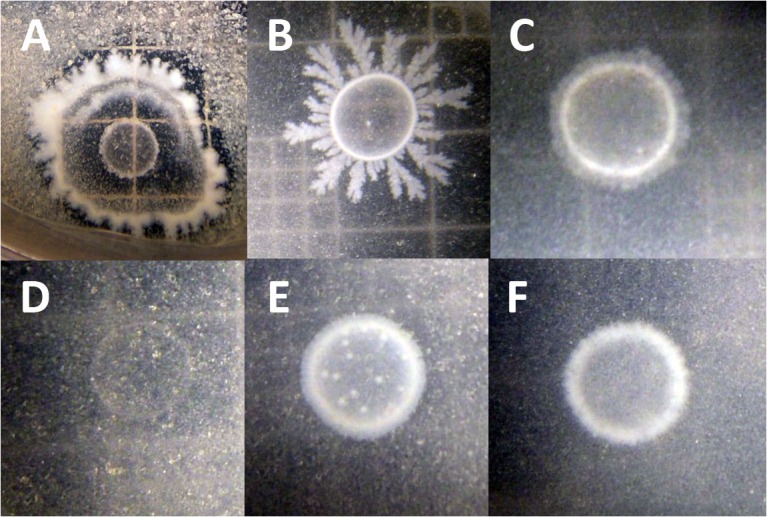
Photographs of chitosan-degrading activity of **(A)** *Paenibacillus tundrae* 47 (positive control) and **(D)** *Kocuria rosea* 245 (negative control). The experimentals are **(B)** *B. subtilis* 30VD-1; **(C)** *B. simplex* 30N-5; **(E)** *B. simplex* 237; **(F)** *B. simplex* 11. Bar, 10 μm.

To test whether *B. simplex* could utilize chitin, the bacteria were grown on colloidal chitin-containing plates (Kuddus and Ahmad, 2013). **Figure 3A** shows that the positive control *P. tundrae* 47 grew robustly on colloidal chitin-containing medium and not only produced a halo indicating chitinolytic activity, but also developed a central clearing of the colony as seen in studies on chitinolytic and chitin deacetylase-producing bacteria (Kaur et al., 2012). Although neither the *B. simplex* strains nor *B. subtilis* 30VD-1 developed obvious halos around the peripheries of their colonies, they all grew on the colloidal chitin-containing medium, especially *B. subtilis* 30VD-1. Moreover, the central part of the colony cleared (**Figures 3B,C,E,F**) as described for chitin deacetylase producers (Kaur et al., 2012). In contrast, *Kocuria rosea* 43 did not grow on the colloidal chitin-containing medium. Only an outline of the inoculum and distinct particles of colloidal chitin were observed even after 3 weeks of incubation (**Figure 3D**).

Genes encoding chitin-degrading enzymes such as lysozyme are also present in the *B. simplex* 30N-5 genome. Two genes, one annotated as lysozyme, and the other as lysozyme-like, were detected in all *B. simplex* strains studied (**Figure 2**). A number of protease encoding sequences are also present, and all of the *Bacillus* strains exhibited protease activity on plates (**Supplementary Figure S2**).

### Other Cell Wall-Degrading Enzymes

Two adjacent genes (IMG2517085627 and -628), one of which, gene -627, was annotated as endopolygalacturonase, are present in the *B. simplex* 30N-5 genome. No pectin methyl esterase encoding genes were found in the *B. simplex* genomes. However, the presence of pectinolytic activity in three *B. simplex* strains and *B. subtilis* 30VD-1 on pectin-containing plates strongly suggests that the gene annotated as endopolygalacturonase is active (**Supplementary Figure S2**). Moreover, *B. subtilis* 30VD-1 produced a much larger halo for its colony size than the *B. simplex* strains (**Table 1** and **Supplementary Figure S2**), suggesting that a similar gene sequence is present.

Both *B. simplex* 30N-5 (IMG251083929) and P558 possess a gene annotated as *xylA*, which is 89.7% identical and orthologous to a gene with the same name in *B. solisalsi* CGMCC 1.6854. A gene encoding a presumed beta-xylosidase (IMG2517086168) was detected in the *B. simplex* 30N-5 genome and the other *B. simplex* genomes. This gene was 100% identical to a gene from *Bacillus* sp. Soil745 annotated as alpha-N-arabinofuranosidase, which is known to act on arabinoxylan. In spite of the lack of more definitive genomic data, evidence for xylanase activity by the *B. simplex* strains and *B. subtilis* 30VD-1 was detected in a plate assay (**Table 1** and **Supplementary Figure S2**). Thus, it is likely that one or more of the genes annotated as xylanase are expressed.

### *In planta* Biocontrol Assessment

We used several different methods to study biocontrol. In a pilot experiment using a protocol described by Abd El Daim et al. (2015), we tested scented stock seeds with FOM and FS, and found that both sets of seeds were quickly overrun with fungal hyphae, but more so when treated with *B. simplex* 30N-5 than with *B. subtilis* 30VD-1. Another test was performed on scented stock seedlings inoculated only with *B. subtilis* 30VD-1 and challenged with the fungi. After 14 days, the FOM- and FS-treated plants were severely stunted whereas the FOM- or FS-infected plants treated with *B. subtilis* 30VD-1 were greener and larger, although not as well developed as the untreated controls. When the experiment was terminated and the dry weights measured, the dry weights of *B. subtilis* 30VD-1 treated FOM- and FS-seedlings were statistically higher than the diseased plants albeit not as high as the non-*Fusarium* infected seedlings (**Supplementary Figure S3**). The *B. subtilis* 30VD-1-treatment of the FOM- and FS-infected plants resulted in a 34.6 and 26.3% increase in dry weight compared to the respective FOM and FS-infected control plants. Using the disease severity scale developed by Diener and Ausubel (2005), the reduction in disease severity was 33.3% for both treatments.

**FIGURE 4:**
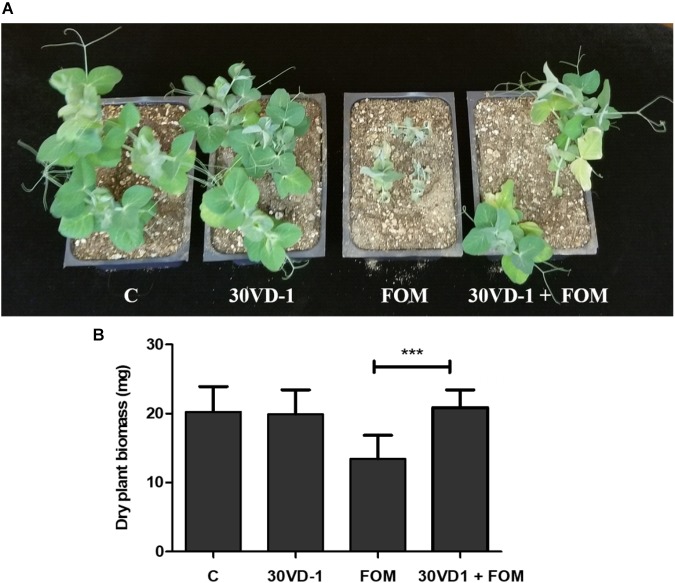
**(A)** Disease severity in pea under *in vivo* conditions. The presented treatments are uninoculated control (C), inoculation with *B. subtilis* 30VD-1 (30VD-1), inoculation with *F. oxysporum* f. sp. *matthioli* (FOM), and co-inoculation of *B. subtilis* 30VD-1 and FOM (30VD-1+FOM). **(B)** Graphical representation of dry biomass of pea plants in response to different treatments in C, 30VD-1, FOM, and 30VD-1+FOM. The asterisks indicate that the co-inoculation of *B. subtilis* 30VD-1 and *Fusarium* had a positive effect on growth. ANOVA univariate analyses were performed and a *post hoc* LSD test was used to identify inoculation treatments with means significantly different from the control at *P* ≤ 0.05.

Because of its larger seed size compared to scented stock, we tested the antagonistic efficacy of the 30VD-1 strain for its ability to control *Fusarium* wilt infection in pea (*Pisum sativum*). The plants raised from 30VD-1 bacterized seeds exhibited reduced disease development and better plant health in FOM-infested soil compared to the non-bacterized controls growing under similar conditions. This observation was supported by a 35.6% increase in dry plant biomass of the 30VD-1+FOM treatment in comparison to FOM inoculation alone (**Figure 4**).

### Growth Inhibition of FOM and FS by BVCs

Many PGP bacilli produce BVCs that inhibit fungal growth (Vespermann et al., 2007; Yuan et al., 2012; Velivelli et al., 2015). We employed a dual plate assay based on previous studies in the literature (Ting et al., 2011) and variations thereof, but the bacteria did not remain confined to their half of the dual plate assay in the time needed to finish the experiment. We next tried the two-Petri dish assay described by Bernier et al. (201l), and first tested pure compounds, which are known to inhibit fungal growth, namely phenol, benzaldehyde, 1-decanol, and 1-dodecanol, in the center plate. The most effective pure chemical tested against FS and FOM was phenol, which completely inhibited fungal growth; the least effective was 2-propanone, which had no effect. Repeated experiments showed that phenol, 1-decanol, 1-dodecanol, and benzaldehyde were inhibitory to fungal growth. These results showed that the fungi responded to the pure chemicals, but when the bacilli alone were used in the experiments, no fungal growth inhibition was observed even after 5 days. The dual plate assay results demonstrated *B. subtilis* 30VD-1 as the most effective biocontrol agent in comparison to the rest of the 3 *B. simplex* strains included in the study. The biocontrol efficacy of the 3 *B. simplex* strains was transient and did not persist for long, so *B. subtilis* 30VD-1 owing to its persistent and effective antagonism toward *Fusarium* spp. was selected for all further *in vitro* and *in vivo* studies.

In the split plate assay, where we grew the fungal cells (FOM/FS) in a liquid medium on one side of the split plate and *B. subtilis* 30VD-1 on the other side, we saw a significant decrease in growth of FOM and FS in response to volatiles. The FOM and FS dry biomass (g) in response to *B. subtilis* 30VD-1 was recorded as 0.496 ± 0.20 and 0.705 ± 0.05 g, respectively, in comparison to the control FOM and FS counterparts, which were 0.935 ± 0.03 and 0.819 ± 0.09 g, respectively. The reduction in FOM and FS biomass following *B. subtilis* 30VD-1 inoculation and incubation was 46.9 and 16.5%, respectively, in comparison to the FOM/FS controls.

### Growth Inhibition of FS and FOM by Culture Filtrates

To determine whether or not a component of the bacterial culture filtrate (CF) was inhibitory to FOM and FS proliferation, we extracted the *B. subtilis* 30VD-1 CF with 1-butanol. *B. subtilis* 30VD-1 CF was mixed with equal amount of 1-butanol, and the partitioned butanol extract on drying was resuspended in sterile distilled water and used as crude butanol extract for fungal growth bioassay. Measurements of fungal growth were made after the fungus in control plate attained full growth. The crude butanol extract caused 40–42% inhibition of FOM and FS growth at 100 μg/ml, and was effective even at a dilution of 1:100 (**Supplementary Figure S4**). Further characterization of the extracted active fraction is in progress.

## Discussion

Plant-growth promoting bacteria have numerous strategies, both direct and indirect, to enhance plant growth and survival. Previously, we observed fungal hyphal thinning in response to coinoculation between *B. simplex* 30N-5 and *F. solani* (Schwartz et al., 2013). In this study, we examined this *B. simplex* strain and strains 11 and 237 in greater detail and analyzed additional *B. simplex* genomes to establish the genetic support for the results we observed. Evidence for genes that encode enzymes in the known chitin degradation pathways in the *B. simplex* and *B. subtilis* 30VD-1 genomes and that are orthologous to genes in the well-known PGP bacilli were found in the *B. simplex* 30N-5 genome. Support for their activity was discovered when the three *B*. *simplex* strains and *B. subtilis* 30VD-1 grew and exhibited a colony phenotype identical to that described by Kuddus and Ahmad (2013) on colloidal chitin-containing plates. This result strongly suggests that the mechanism predicted by the genome analysis – deacetylation of chitin through chitin deacetylase activity and hydrolysis of the deacylated chitin by chitosanase – is functional. Thus, the *in silico* and experimental studies agreed, demonstrating a direct correlation between genotype and phenotype.

We also detected several genes potentially encoding hydrolytic enzymes and found evidence for the synthesis of these enzymes through specific plate assays. The ability to break down fungal cell walls is a defining characteristic of many BCAs, including *Streptomyces galilaeus* CFFSUR B-12, which exhibits antifungal activity against *Mycosphaerella fijiensis*, a plant pathogen, and *F. oxysporum* f. sp. *lycopersici*, which causes tomato wilt (Castillo et al., 2016). Other cell wall-degrading enzymes produced by bacilli include cellulases, pectinases, and xylanases, and genes encoding these hydrolytic enzymes were detected in the *B. simplex* genomes. Bacteria employ hydrolytic enzymes to break down plant cell wall material for utilizable carbon, but endophytes also use these enzymes to enter plant tissues. *B. simplex* 11 is an endophyte based on its isolation from dead root material (Kaplan et al., 2013), and other *B. simplex* strains are known to be endophytes in maize (Xia et al., 2015). In addition, endophytic bacteria have been shown to control *Fusarium*-induced plant diseases (Shahzad et al., 2017).

The BVC 2,3-butanediol and other volatiles promote growth in Arabidopsis in response to pathogens (Ryu et al., 2003). Both *B. subtilis* GB03 and *B. amyloliquefaciens* IN937a synthesize 3-hydroxy-2-butanone (acetoin), which gives rise to 2,3-butanediol, the proposed inducer. In our previous study (Maymon et al., 2015), we could not find in the genome of *B. simplex* 30N-5 the gene *alsD*, which encodes alpha-acetolactate dehydrogenase, the enzyme required for the decarboxylation of acetolactate into acetoin. Moreover, *B. subtilis* 30VD-1 and the three strains studied here do not produce acetoin, which was also the case for *B. simplex* M3-4 (Velivelli et al., 2015). However, other BVCs produced by bacteria protect plants from biotic stress, and previous work demonstrated that *B. subtilis* strains produce a number of them (Fiddaman and Rossal, 1993; Chaurasia et al., 2005; Farrag et al., 2006; Arrebola et al., 2010). *B. simplex* and *B. subtilis* are known to synthesize 2-propanone, phenol, 1-decanol, 2-undecanone, 1-decanol, 1-dodecanol, methyl-tetradecanoate, and 2-ethyl-3,5-dimethylpyrazine, and many other microbial VOCs (Velivelli et al., 2015; Lemfack et al., 2018). Gu et al., 2007 found that *B. simplex* BVCs from six distinct isolates killed almost 100% of the parasitic nematodes *Panagrellus redivivus* and *Bursaphelenchus xylophilus*. When pure compounds were employed, these authors further determined that the BVCs that elicited the highest nematode mortality were phenol, 1-decanol, 2-nonanone, 2-undecanone, phenylethanone, cyclohexene, and benzaldehyde.

Based on the fact that several of the BVCs resulting in nematode death overlap with the antifungal BVCs used by *B. amyloliquefaciens* NJN-6 (Yuan et al., 2012), we expected that the *Bacillus* strains in our study would synthesize antifungal BVCs and inhibit the growth of FOM and FS. Our experiments using the dual plate assays at first suggested that the *B. simplex* strains and *B. subtilis* 30VD-1 inhibited FOM growth, but repetitions under similar conditions showed no effect on fungal colony diameter. Only in the cases where pure chemical compounds were employed, did we see a dramatic effect. However, because the cultures were touching after 7 days, we decided to employ more restrictive experimental regimes to separate the bacteria and the fungi. The split plate method, when the cultures were grown in liquid instead of solidified medium, led to a reduction in dry fungal biomass in comparison to the control plates. Zhang et al. (2014) noted that BVCs were less effective overall than actual bacteria in inhibiting the growth of certain fungi including *F. oxysporum*. Our results suggested that contact was necessary for complete FOM- and FS-growth inhibition leading us to test whether CF extracts were involved in mycelial growth inhibition, which they were.

In nature, many factors influence inter-organism communication. Soil bacteria that associate with plants often live in nutrient-poor soil, but most studies on plant-growth promotion and biocontrol are performed under close-to-ideal nutrient conditions. Also, bacteria in nature live in consortia communicating with one another as well as with eukaryotic organisms and other bacteria. Data show that either combining PGPB or co-inoculating PGPB and non-pathogenic fungi serve as more effective BCA than a single bacterial strain (Jetiyanon and Kloepper, 2002; de Boer et al., 2003; Schmidt et al., 2015; and references therein). Also, as indicated for BVCs, it is likely that cooperation is common between BCA and plants (Baffoni et al., 2015), but as yet few of the factors involved in this cooperativity and/or host specificity have been thoroughly investigated. As a consequence, whether the transition occurs with the same efficacy from laboratory and greenhouse conditions, where positive responses are observed, to field conditions is a major challenge (Compant et al., 2005).

More challenges will arise as attempts are made to change from the use of synthetic fertilizers and pesticides to sustainable agricultural practices whereby beneficial soil microbes promote plant growth and protect plants from predator and pathogen attack. In addition, PGPB enable plants to obtain limiting nutrients, such as Fe, P, and N, which also improve plant health. This effort will require a combination of genomic, biochemical, and experimental analyses as well as testing pairwise and more complex combinations.

## Materials and Methods

### Organisms Investigated and Inoculum Preparation

Several of the bacteria and fungi examined in this study were described previously (Schwartz et al., 2013). *B. simplex* 237, isolated from soil under the canopy of *Zygophyllum dumosum*, and *B. simplex* 11, isolated from surface-sterilized, dried *Z. dumosum* root tissue (Kaplan et al., 2013), were also investigated (**Table 2**). *B. amyloliquefaciens* subsp. *plantarum* FZB42 and *B. subtilis* GB03, obtained from the *Bacillus* stock center, were used as controls in several of the physiological assays. For the chitin-degradation assay, the procedure used by Kaplan et al. (2013) was followed. *P. tundrae* 47 and *Kocuria rosea* 43 were used as positive and negative controls, respectively. Details about the strains are presented in **Table 2**.

**Table 2:** Strain list.

Name	Relevant characteristics	Source/Reference
*B. simplex* 30N-5	Isolated from the rhizosphere of a *Podocarpus nagi* tree, Mildred E. Mathias Botanical Garden, UCLA	Schwartz et al., 2013
*B. simplex* 237	Isolated from soil under the canopy of *Zygophyllum dumosum*, Negev Desert	Kaplan et al., 2013
*B. simplex* 11	Isolated from dried root tissue of *Z. dumosum*, Negev Desert	Kaplan et al., 2013
*Bacillus subtilis* 30VD-1	Isolated from soil adjacent to *Brahea edulis* (Arecaceae; Guadalupe Palm), a tree indigenous to Guadalupe Island in Mexico, Mildred E. Mathias Botanical Garden, UCLA	Schwartz et al., 2013
*Paenibacillus tundrae* 47	Isolated from *Z. dumosum* rhizosphere, Negev Desert	Kaplan et al., 2013
*Kocuria rosea* 245	Isolated from *Z. dumosum* rhizosphere, Negev Desert	Kaplan et al., 2013

The *B. simplex, B. subtilis*, and *B. amyloliquefaciens* strains were grown overnight at 30°C on agar-solidified Tryptone Yeast extract (TY) or Luria-Bertani (LB) medium. The fungi, *Fusarium oxysporum* f. sp. *matthioli* (FOM), *F. oxysporum* f. sp. *conglutinans* (FOC), and *F. solani* (FS) were maintained on Potato dextrose agar (PDA), and based on experimental requirements were grown on PDA or solidified V8 medium (Miller, 1955) at 30°C for a week prior to their use in the anti-fungal assays.

### *In vitro* Bacteria–Fungus Interaction Assays

#### Qualitative Evaluation of Bacterial Antagonism due to Diffusible and Volatile Compounds

The testing of biocontrol efficiency due to diffusible compounds was performed by growing the pathogen (FOM/FOC/FS) and test bacteria on V8 agar using a dual culture technique. Briefly, the bacterial strains were grown overnight in TY liquid medium and then after centrifugation to eliminate the culture medium, were diluted with sterilized Millipore water to an OD_600_ value between 0.35 and 0.40. Fifty microliters of the bacterial suspensions were streaked in a line on V8 agar in conventional Petri dishes 1 cm from the plate edge. Fungal plugs 5 mm in diameter were then placed on the agar 2.5 cm away from the bacterial streak either immediately after the bacteria were plated (for *B. subtilis* 30VD-1 and the water control) or after 24 h (for the slower growing *B. simplex* strains). The plates were incubated at 30°C. The fungal radii were measured both toward and away from the bacterial streak every 24 h for 7 days at 15°, 10°, 4°, 0°, −4°, −10°, and −15° from the perpendicular formed by the plug and bacterial line. The radii were measured from the center of the fungal plug until the point where the region of highest hyphal density began to break down. The percent fungal growth inhibition was calculated using the equation: [Growth Inhibition = (*F*_Bacteria_–*F*_Water_)/*F*_Water_], where *F*_Bacteria_ is the radius of fungal growth toward the bacteria and *F*_Water_ is the radius of fungal growth toward the water in the control. Each experiment was repeated three or more times, with 10 replicates for each treatment.

### Hydrolytic Enzyme Assays

Fifteen to twenty microliters of an overnight bacterial culture were spotted onto LB, TY, or specialized media for testing hydrolytic enzyme activity. After 3–4 days of incubation, the three *B. simplex* strains and *B. subtilis* 30VD-1 were examined on the assay plates. For detecting cellulase and xylanase activity, a procedure modified from Teather and Wood (1982) was used in which the overlay medium was eliminated and carboxymethylcellulose (CMC) was incorporated directly in the plate medium. Pectinase activity was detected on a pectin (either apple or citrus peel)-containing medium as described by Namasivayam et al. (2011) after 5 days of incubation. The pectin plates were left unstained or visualized with 0.05% ruthenium red staining for 20 min followed by several rinses with distilled water.

To detect the activity of chitin-degrading enzymes, the colloidal chitin-containing medium described by Kuddus and Ahmad (2013) was used. Clear zones around or within the colonies are considered evidence of chitinase activity in a plate containing colloidal chitin (Kaur et al., 2012) whereas growth of the colony along the edges on the medium coupled with a central clearing are diagnostic of chitin deacetylase activity (Kaur et al., 2012). The experiment was repeated twice with 4–5 replicates per experiment.

Protease activity was determined using skim milk agar medium, which contained (per liter): 5 g pancreatic digest of casein, 2.5 g yeast extract, 1 g glucose, 7% skim milk solution, and 15 g of agar (Naik et al., 2008). Bacterial cells were spot-inoculated and after 2 days incubation at 28°C, the plates were flooded with Coomassie Brilliant Blue (0.25% w/v) dissolved in methanol:acetic acid:water (5:1:4 v/v) for 10 min to enhance the visibility of halos around the bacterial colonies. Destaining with methanol:acetic acid:water was performed to ensure better contrast before taking pictures of the assay plates. Photographs were taken on an Illuma System Light Control platform with a Canon PowerShot ELPH350HS camera. Each experiment was repeated three times with 3 or more colony replicates.

### Genome Analysis

The draft and finished genomes used for the analysis of the PGPB (Maymon et al., 2015) were utilized in this study, except that the genomes of *B. simplex* BA2H3 (NCBI Accession number: NZ_KN360955) and *B. simplex* 558 (NCBI Accession number: NZ_CCXW01000001) were also included, as well as *B. subtilis* 30VD-1 (IMG ID number 2786546174) and the two *Paenibacillus* genomes from the earlier paper were eliminated. A group of manually annotated genes from the *B. simplex* 30N-5 genome, which were likely to be involved in biocontrol based on the literature, served as the reference genes (set at 100%) to query known PGP *Bacillus* species in the Joint Genome Institute IMG/ER (Markowitz et al., 2012) or NCBI^1^ databases for blastp homologs. As in our previous study (Maymon et al., 2015), the *K*-means clustering algorithm established the species order shown in **Figure 2**. The blastp searches were filtered to include conservative alignments of *e* ≥ 10^−5^ and sequence identities of 50% or higher. Identities greater than 50% were indicated by different shades of orange in **Figure 2** whereas those less than 50%, which were retained because of potential gene homology, were shaded blue. Genes that were missing either because the genome is unfinished or because the gene was not found are indicated as “n.d.” (not detected) in unshaded cells of the table.

### Antifungal Assays for BVCs

Bacterial strains were evaluated for their volatile-mediated antifungal activity against FOM and FS using the centrally partitioned Petri dishes (85 mm × 15 mm) with (Bernier et al., 2011) or without physical contact between the two microorganisms grown on either side. The larger partition was filled with 10 ml LB agar for the growth of bacteria and the smaller one with 10 ml potato dextrose broth (PDB) for the fungal counterpart. One hundred microliters of a 48 h grown bacterial suspension containing approximately 10^9^ CFU/mL were spread onto the LB agar and a fungal spore suspension containing 10^6^ spores/mL resuspended in water was inoculated into the PDB medium. The plates were then sealed with Parafilm and incubated in the dark for 7 days. The controls were uninoculated LB agar and PDB in the separate partitions.

Following incubation at room temperature in the dark for 7 days, the antifungal effect of bacterial volatiles on the fungal pathogen was recorded in terms of reduction in dry fungal biomass as compared to the control. For fungal dry biomass observations, the PDB compartment was emptied onto a pre-weighed Whatman filter paper and dried at 60°C. Reductions in dry fungal biomass readings were measured and calculated as percentages.

### *In vivo* Biocontrol Assays

Seeds of *Matthiola longipetala* ssp. *bicornis* (scented stock), obtained from Baker Creek Heirloom Seed Company, Mansfield, MO, United States, were surface-sterilized by immersing them in 95% ethanol for 1 min, followed by three rinses with sterile distilled water. For the treatments, seeds were bacterized for 3 h by imbibing them in a bacterial suspension, which had been grown for 48 h to contain approximately 10^9^ CFU/ml. Seeds imbibed in LB medium served as the control. The seeds were placed into sterile OPTILUX^TM^ Petri dishes (100 mm × 20 mm) containing complete Hoagland’s medium solidified with 1% agar until germination. At that time, healthy seedlings were gently uprooted from the agar and dipped for 30 min into distilled water, or into an aqueous suspension of *B. subtilis* 30VD-1, or inoculated with a spore suspension of FOM or FS (10^6^ spores/ml), or both *B. subtilis* 30VD-1 and FOM or FS. The control and treated seeds were then replanted onto 0.5% Hoagland’s agar. The seedlings were incubated for 3 days in the dark at 28°C and then exposed to light until 15 days after the initiation of experiment.

Based on an assay developed by Diener and Ausubel (2005) for Arabidopsis, the disease severity index was modified for the stock and pea experiments. Healthy plants classified as 0 or 1 were green, but plants rated with an index of 1 had smaller leaves than the healthy 0-ranked plants. Stock plants showing moderate yellowing and wilting with less developed leaves were given a disease severity index of 2 whereas plants displaying additional disease symptoms such as acute yellowing, wilting, and an even greater reduction in leaf area were considered as having an index level of 3. Plants with a disease severity index of 4 were highly stunted whereas dead or near dead plants were categorized as index 5. Plant dry weight measurements were taken. The plants were gently uprooted and placed into pre-weighed microcentrifuge tubes, air-dried and then measured on an analytical balance. There were 10 replicates for each experiment, which was repeated four times.

Because FOM has a broad host range, pea (*Pisum sativum*) was used as a test plant for pathogenicity and protection by *B. subtilis* 30VD-1. Seeds of pea (*var*. Little Marvel) were surface-sterilized and bacterized as described earlier. Seeds imbibed in bacteria-free medium were used as a control. Pathogen challenge was performed by mixing the spore suspension in soil such that the spore count was 10^5^ spores/g of soil. Thus, treatments utilized in the study were the control, 30VD-1 treated, FOM-treated, and 30VD-1+FOM-treated. Six replicates of each treatment, with five plants in each pot, were maintained. The experiment was conducted in a Conviron plant growth chamber. *Fusarium* wilt assessment was conducted after 45 days according to the scale described above. Plant dry weight measurements were made after drying the plants in a 60°C oven.

Photographs of the plates and the plants were taken with a Canon PowerShot ELPH350HS camera.

### Data Analysis

The statistical analysis of the data was performed using SPSS software, version 21 (SPSS Inc., Chicago, IL, United States). After collection of the fungal growth parameters, or distances to the bacterial culture, the data were processed with one-way analysis of variance (ANOVA). A *post hoc* Fisher’s LSD test was used to identify treatments with means significantly different from the water control at *P* ≤ 0.05.

## Author Contributions

AH, NK, and PM-H were involved in planning and execution of the research, analysis and interpretation of the data, and writing the manuscript. TI optimized and performed the biocontrol assays along with NK, PM-H, and NN. MM performed the *in vitro* assays and helped in writing and editing the manuscript. AH did the genome analysis and ES and DK contributed strains and intellectual support. EH verified the identity of all the strains and edited the manuscript.

## Conflict of Interest Statement

The authors declare that the research was conducted in the absence of any commercial or financial relationships that could be construed as a potential conflict of interest.

## Footnotes

1 http://www.ncbi.nlm.nih.gov

## Supplementary Material

FIGURE S1 Phase contrast micrographs of FOM; 40× magnification. Fungal samples were collected from control **(A)** and dual culture plates containing FOM and *B. subtilis* 30VD-1 **(B)**. Arrows indicate hyphal distortions and abnormal bulbous structures, and the dotted arrow points to the extrusion of cytoplasmic contents in response to 30VD-1 treatment.

FIGURE S2 Agar-plate based clearing assays for detecting the production of cellulase **(A–D)**, protease **(E–H)**, pectinase **(I–L)**, xylanase **(M–P)**; Column 1 denotes *B. simplex* 30N-5; column 2, *B. simplex* 237; column 3, *B. simplex* 11; and column 4, *B. subtilis* 30VD-1. The colonies in each row are of similar sizes to compare variations in halo diameter.

FIGURE S3 **(i)** Disease severity in scented stock seedlings. The presented treatments are uninoculated control (C), inoculation with *B. subtilis* 30VD-1 (30VD-1), inoculation with *F. oxysporum* f. sp. *matthioli* (FOM), co-inoculation of *B. subtilis* 30VD-1 and FOM (30VD-1+FOM), inoculation with *F. solani* (FS) and co-inoculation of *B. subtilis* 30VD-1 and FS (30VD-1+FS). **(ii)** Graphical representation of dry weights of scented stock seedlings after different treatments in C, 30VD-1, FOM, 30VD-1+FOM, FS, and 30VD-1+FS. Values are mean of 10 plants ± SD. Experiment was repeated 4 times. Statistical significance of biomass data was validated using one-way ANOVA with Tukey’s *post hoc* test and multiple comparison procedure. Different letters represent values that differ significantly, *P* < 0.05.

FIGURE S4 Biocontrol potential of a crude butanol extract of *B. subtilis* 30VD-1. The treatments are D (*B. subtilis* 30 VD-1’s crude butanol extract at 100 μg/ml concentration). 1:10, 1:50, and 1:100 are 10-, 50-, and 100-fold dilutions of the crude butanol extract of the *B. subtilis* 30VD-1 culture filtrate.
