# Supplementary figures and images for: Antifungal Activity of Bacillus Species Against Fusarium and Analysis of the Potential Mechanisms Used in Biocontrol
Source: Front Microbiol. 2018 Oct 2;9:2363. doi: 10.3389/fmicb.2018.02363 (PMC6176115; doi:10.3389/fmicb.2018.02363)

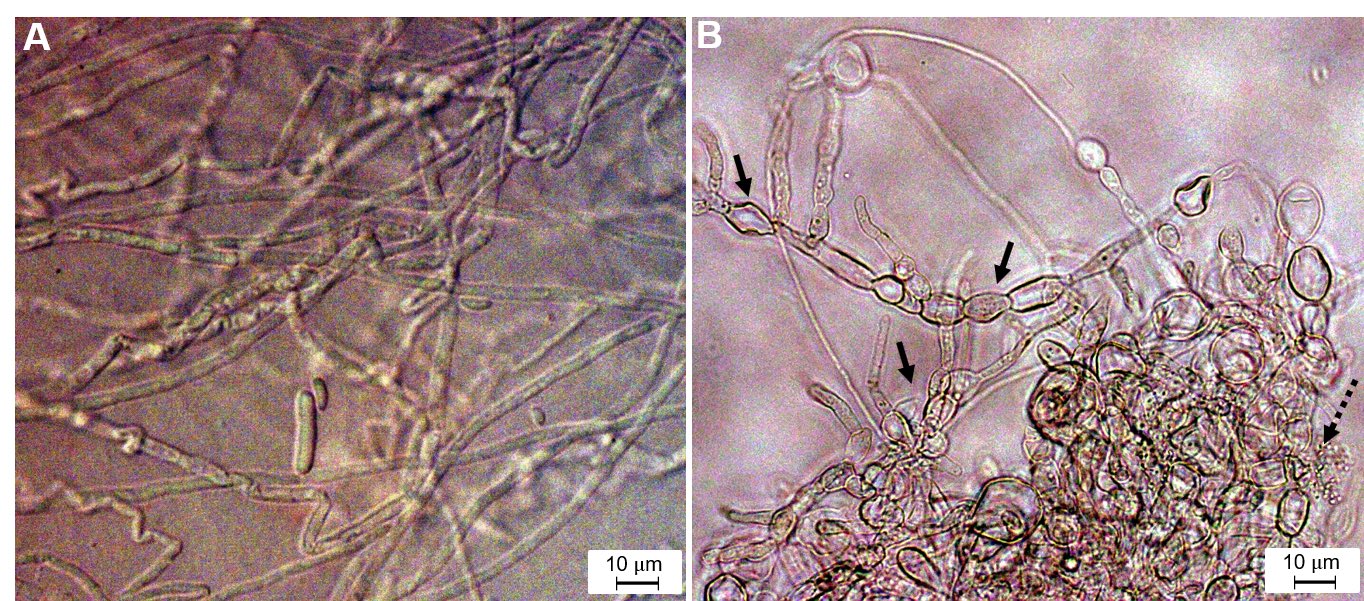

Supplement: FIGURE S1 — Phase contrast micrographs of FOM; 40× magnification. Fungal samples were collected from control (A) and dual culture plates containing FOM and B. subtilis 30VD-1 (B). Arrows indicate hyphal distortions and abnormal bulbous structures, and the dotted arrow points to the extrusion of cytoplasmic contents in response to 30VD-1 treatment. [file Image_1.JPEG]

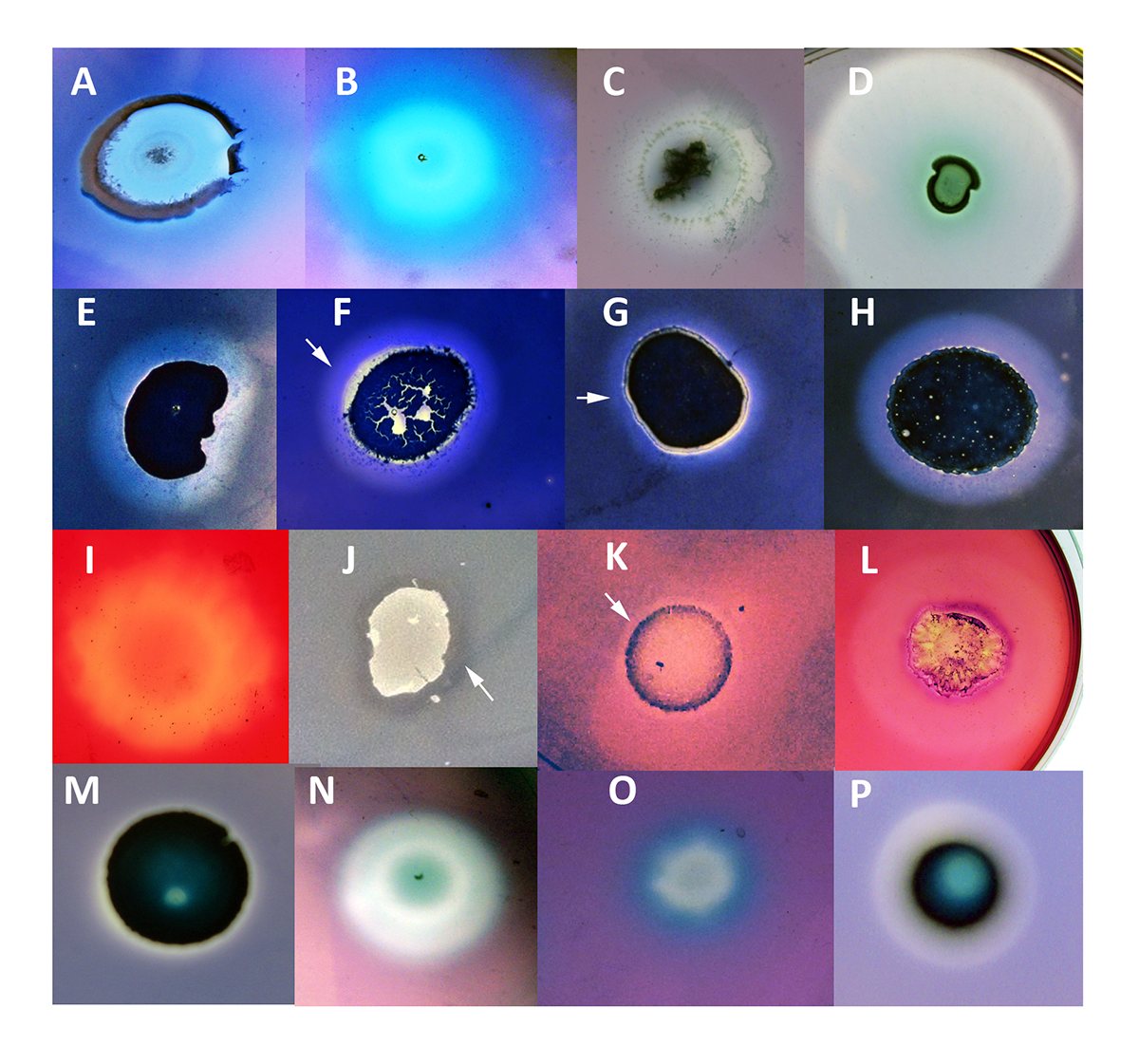

Supplement: FIGURE S2 — Agar-plate based clearing assays for detecting the production of cellulase (A–D), protease (E–H), pectinase (I–L), xylanase (M–P); Column 1 denotes B. simplex 30N-5; column 2, B. simplex 237; column 3, B. simplex 11; and column 4, B. subtilis 30VD-1. The colonies in each row are of similar sizes to compare variations in halo diameter. [file Image_2.TIF]

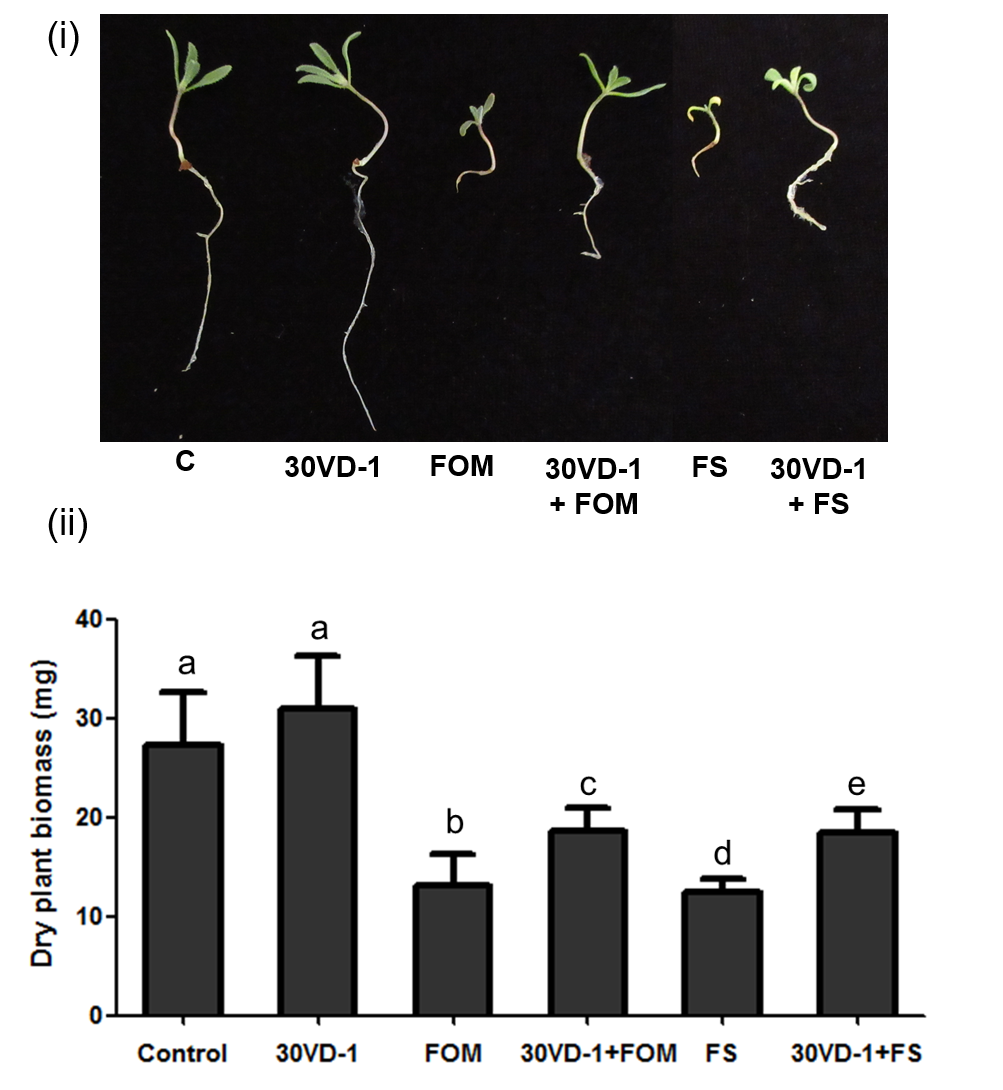

Supplement: FIGURE S3 — (i) Disease severity in scented stock seedlings. The presented treatments are uninoculated control (C), inoculation with B. subtilis 30VD-1 (30VD-1), inoculation with F. oxysporum f. sp. matthioli (FOM), co-inoculation of B. subtilis 30VD-1 and FOM (30VD-1+FOM), inoculation with F. solani (FS) and co-inoculation of B. subtilis 30VD-1 and FS (30VD-1+FS). (ii) Graphical representation of dry weights of scented stock seedlings after different treatments in C, 30VD-1, FOM, 30VD-1+FOM, FS, and 30VD-1+FS. Values are mean of 10 plants ± SD. Experiment was repeated 4 times. Statistical significance of biomass data was validated using one-way ANOVA with Tukey’s post hoc test and multiple comparison procedure. Different letters represent values that differ significantly, P < 0.05. [file Image_3.TIF]

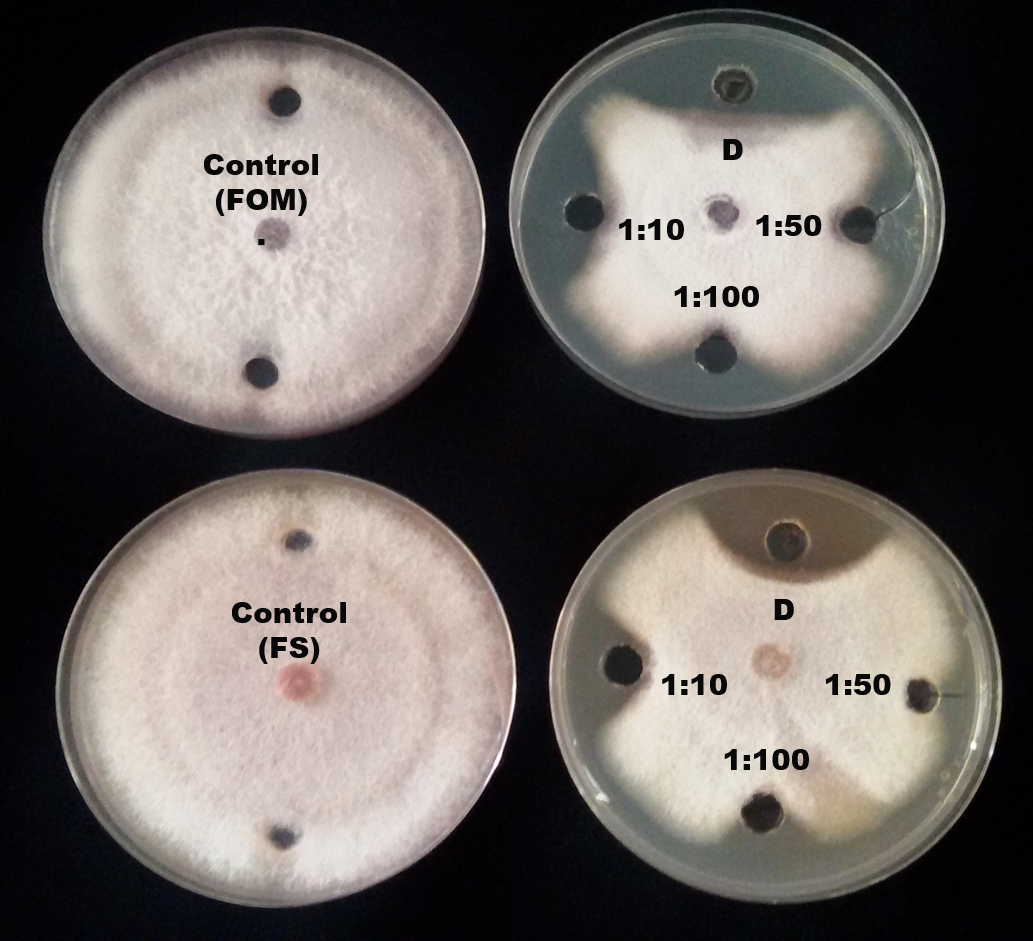

Supplement: FIGURE S4 — Biocontrol potential of a crude butanol extract of B. subtilis 30VD-1. The treatments are D (B. subtilis 30 VD-1’s crude butanol extract at 100 μg/ml concentration). 1:10, 1:50, and 1:100 are 10-, 50-, and 100-fold dilutions of the crude butanol extract of the B. subtilis 30VD-1 culture filtrate. [file Image_4.TIF]
